# Cold inducible RNA binding protein upregulation in pituitary corticotroph adenoma induces corticotroph cell proliferation via Erk signaling pathway

**DOI:** 10.18632/oncotarget.7037

**Published:** 2016-01-27

**Authors:** Fangfang Jian, Yufan Chen, Guang Ning, Wei Fu, Hao Tang, Xiao Chen, Yao Zhao, Lili Zheng, Sijian Pan, Weiqing Wang, Liuguan Bian, Qingfang Sun

**Affiliations:** ^1^ Department of Neurosurgery, Ruijin Hospital, Shanghai Jiaotong University School of Medicine, Shanghai, China; ^2^ Department of Neurosurgery, Changzheng Hospital, The Second Military Medical University, Shanghai, China; ^3^ Department of Neurosurgery, Shanghai Pituitary Tumor Center, Huashan Hospital, Shanghai Medical College, Fudan University, Shanghai, China; ^4^ Department of Endocrine and Metabolic Diseases, Ruijin Hospital, Shanghai Jiaotong University School of Medicine, Shanghai, China; ^5^ Department of Neurosurgery, Ruijin Hospital, Luwan Branch, Shanghai Jiaotong University School of Medicine, Shanghai, China

**Keywords:** Cushing's disease, cold inducible RNA binding protein, Erk pathway

## Abstract

Cushing's disease is caused by pituitary corticotroph adenoma, and the pathogenesis of it has remained obscure. Here, we showed that cold inducible RNA binding protein (CIRP) was markedly elevated in corticotroph tumors. Forced overexpression of CIRP in murine AtT20 pituitary corticotroph cell line increased corticotroph precursor hormone proopiomelanocortin (POMC) transcription, ACTH secretion and cellular proliferation. *In vivo*, CIRP overexpression promotes murine corticotroph tumor growth and enhances ACTH production. Mechanistically, we show that CIRP could promote AtT20 cells proliferation by inducing cyclinD1 and decreasing p27 expression *via* Erk1/2 signaling pathway. Clinically, CIRP overexpression is significantly correlated with Cushing's disease recurrence. CIRP appears to play a critical tumorigenesis function in Cushing's disease and its expression might be a useful biomarker for tumor recurrence.

## INTRODUCTION

Cushing's disease (CD), caused by an adenoma arising from pituitary corticotroph cells, is a rare, potentially life-threatening endocrinopathy with an incidence of 0.7∼2.4/million/year [1, 2]. It caused a series of complications, such as hypertension, obesity, dyslipidemia, diabetes mellitus, cardiovascular disease, osteoporosis, infections and psychiatric disorders, which associated with increased morbidity and mortality if not appropriately treated [3, 4]. Transsphenoidal surgery has been established as the first-line treatment of choice, with remission rates of 65-90% when an expert pituitary surgeon operates [5], hypercortisolism, however, persists or recurs in an important subset of patients [6, 7]. Repeat surgery is an option in patients with persistent or recurrent CD, but the remission rate is lower and the risk for hypopituitarism is considerably high [8]. Novel therapeutic approaches that directly target pituitary tumor growth and/or ACTH secretion remains a major challenge. Improved understanding of mechanisms underlying tumor growth and dysregulated ACTH secretion may lead to molecular targeted therapy.

Cold inducible RNA binding protein (CIRP, also called Cirbp) was originally identified in the testis as the first mammalian cold shock protein [9]. CIRP belongs to the glycine-rich RNA-binding protein family, which possesses an amino-terminal RNA recognition motif and a carboxyl-terminal glycine-rich domain. It is constitutively expressed at low levels in various tissues and is induced by cellular stresses such as hypothermia, UV irradiation and hypoxia [10-12]. CIRP contributes to the maintenance of stemness of neural stem cells [13]. It is suggested to participate in biological rhythms and required for high-amplitude circadian gene expression [14]. In addition, CIRP mRNA and protein was upregulated in several different cancers and it could promote oncogenesis [15, 16]. However, the biological role of CIRP in corticotroph adenoma has remained to be elucidated.

Here, we demonstrate that CIRP expression is markedly increased in human corticotroph tumors as compared to normal pituitary tissues. We further demonstrate that CIRP is a potent regulator of proopiomelanocortin (POMC) transcription, ACTH secretion and corticotroph cell proliferation *in vitro* and *in vivo*. These results elucidate a mechanism which we believe to be novel underlying corticotroph tumorigenesis, and it also provide a rationale for CIRP as a potential therapeutic target to abrogate corticotroph tumor growth and/or ACTH hypersecretion. This study represents the first report of the functional link between CIRP and corticotroph adenoma. Also, this is one of the first papers on the cellular response to stress in Cushing adenomas, which is emerging as the main biological process affected in these tumors.

## RESULTS

### CIRP is overexpressed in human corticotroph adenoma

**Figure 1:**
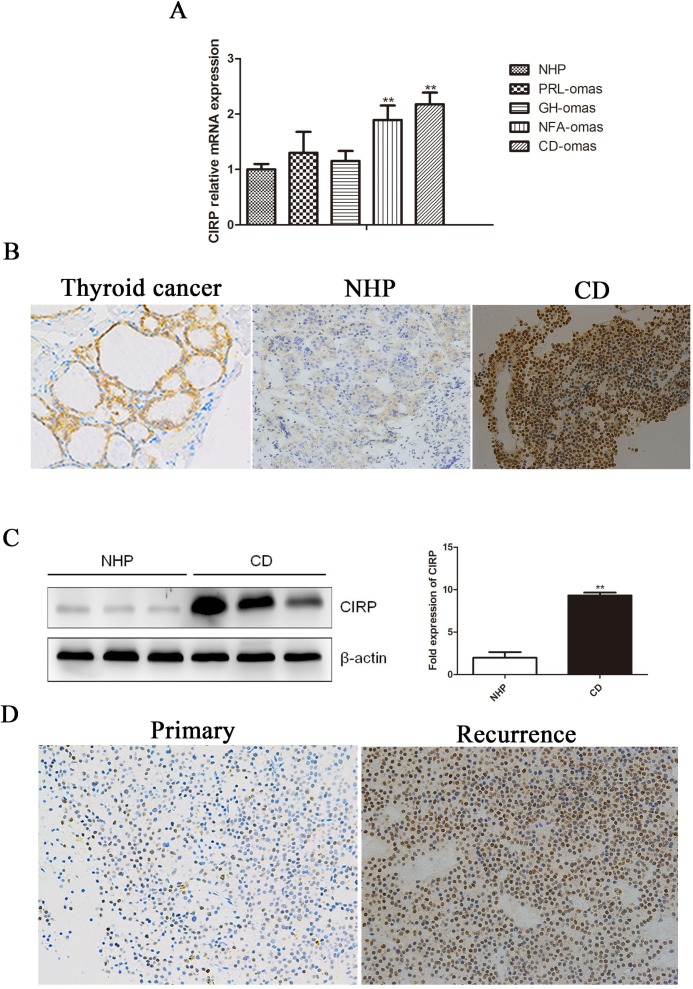
Human corticotroph adenoma shower higher CIRP expression **A.** The expression of CIRP mRNA in ACTH (*n* = 17), GH (*n* = 8), PRL (*n* = 6), nonfunction adenoma (*n* = 7) and normal pituitary tissue (*n* = 7). **B.** Immunohistochemical staining of CIRP in a representative normal pituitary and corticotroph adenoma. Original magnification, ×200. Thyroid cancer serves as a positive control. **C.** Immunoblot analysis of CIRP protein levels in normal pituitary samples and CD. Each lane represents one case. Quantification of the CIRP expression relative to β-actin in the same tissues shown on the right. **D.** Immunohistochemical staining for CIRBP in the matched primary (A)-recurrent (B) samples.

CIRP mRNA was highly expressed in corticotroph adenoma compared to the normal pituitary tissue (Figure 1A) (Figure S1). There was a statistically significant difference in CIRP expression between corticotroph adenoma and normal pituitary tissue (*P* < 0.01). Moreover, the highly expressed CIRP mRNA in CD was also validated by our RNA-seq data (Data not shown). After the identification of pituitary adenoma using hematoxylin and eosin staining and pituitary hormone immunohistochemistry, CIRP protein expression was investigated by immunohistochemical analysis in corticotroph adenoma and normal pituitary samples. Immunoreactivity of CIRP protein was observed in the nucleus of tumorous corticotrophs (Figure 1B), while rare CIRP expression was detected in normal pituitary cells. And the increased CIRP protein levels in tumors from CD patients were further verified by immunoblotting analysis (Figure 1C). In addition, CIRP mRNA was significantly increased in nonfunctioning adenoma (*P* < 0.01); its expression level in somatotroph adenomas and prolactinoma was not changed.

### CIRP overexpression in corticotroph adenoma is associated with recurrence

High levels of CIRP were detected in 23 of the whole Cushing's cohort (23/45, 51.1%). We then further explored whether patients with different CIRP levels display distinct clinical features (Table 1). Six (6/18) patients with higher CIRP expression relapsed compared to that in one (1/20) with negative CIRP (33.3% *vs* 5%; *P* < 0.05). Moreover, in patients with recurrence after TSS, CIRP expression levels in sample from secondary surgery were significantly higher than that from the initial ones (Figure 1D). However, there is no significant correlation between CIRP expression and gender, age, midnight cortisol, urinary free cortisol or plasma ACTH level.

**Tabel 1:** Clinical characteristics of the 45 CD patients according to the CIRP status

characteristics	Negative *N* = 22	Positive *N* = 23	*P* value
Female no. (%)	18 (81.8%)	20 (87.0%)	0.700
Age at diagnosis (yrs)	41.8±10.0	40.0±14.8	0.467
Plasma ACTH (pg/ml)	159.5±146.9	155.1±198.2	0.212
Midnight serum cortisol (μg/dl)	21.7±10.7	21.2±8.3	0.892
Urinary free cortisol (μg/24h)	551.5±369.8	562.9±398.2	0.820
Remission	20 (90.9%)	18 (78.3%)	0.414
Postoperative recurrence	1 (5%)	6 (33.3%)	0.038

### CIRP induces POMC expression and stimulates ACTH secretion in AtT20 cells

**Figure 2:**
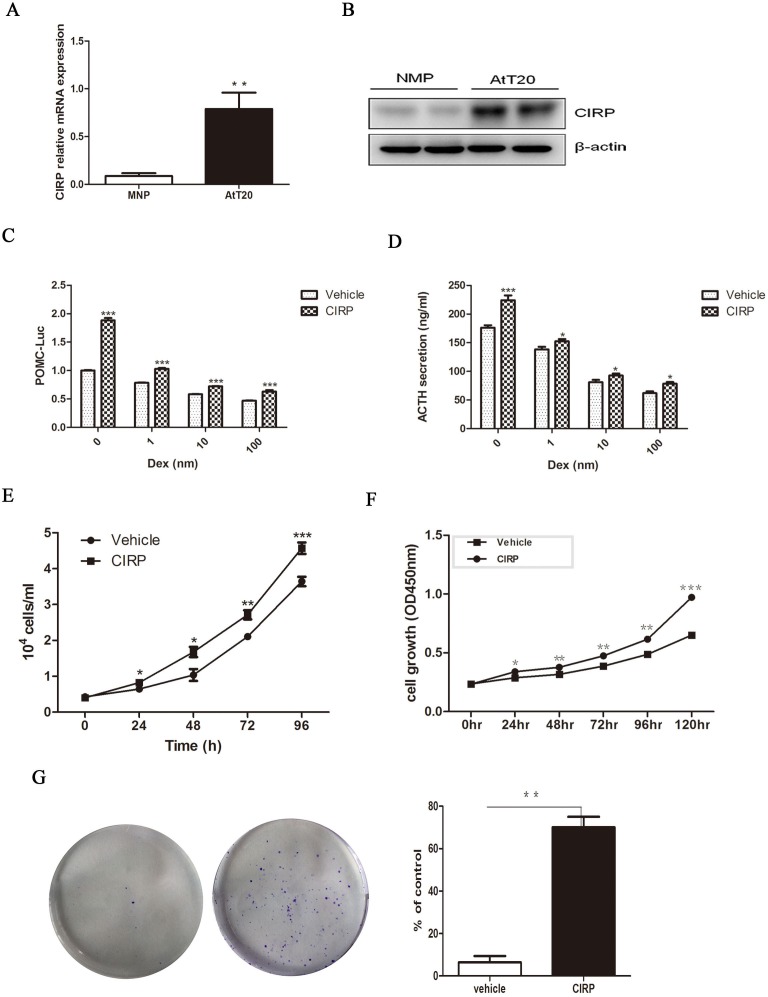
CIRP promote ACTH secretion and proliferation in AtT20 cells **A.** The expression of CIRP mRNA in AtT20 cells and normal mouse pituitary, tested by qRT-PCR. **B.** Western blot of CIRP protein level in AtT20 cells and normal mouse pituitary (NMP). **C.** CIRP effects on baseline and Dex-depressed POMC transcription, Dex: dexamethasone. **D.** CIRP effects on baseline and Dex-depressed ACTH secretion. **E.** cell count (time course). **F.** Effects of CIRP on AtT20 cells proliferation. **G.** Formation of AtT20 colonies after CIRP treatment and quantitative representation of the colonies formed is shown on the right. * *P* < 0.05, **P < 0.01.

The marked increase of CIRP expression in corticotroph tumors as compared with normal pituitary tissue, combined with its correlation with recurrence, suggested upregulation of the CIRP might be involved in the initiation and progression of corticotroph adenomas. As human pituitary tumors do not survive in long-term culture and no human pituitary corticotroph cell line was available, we used murine AtT20 pituitary corticotroph cell line to examine the role of CIRP in corticotroph tumorigenesis. First, we evaluated CIRP expression in AtT20 cells; CIRP mRNA and protein levels were higher in AtT20 cells as compared to normal mouse pituitary tissue (Figure 2A, 2B). To investigate the functional role of CIRP in tumorous corticotrophs, we generated stably transfected AtT20 cells using a retroviral vector containing CIRPWT cDNA, or empty vector (EV). The efficiency of overexpression was evaluated by qRT-PCR and immunoblotting analysis, which showed abundant CIRP mRNA and protein expression in CIRP-transfected AtT20 cells, but not in vector-transfected cells (Figure S2). Dexamethasone-treatment of AtT20 cells, transiently transfected with POMC-promoter, resulted in 11.5%-53.0% decrease in POMC-transcription. CIRP treatment alone, or prior to dexamethasone treatment, promoted basal and dexamethasone-mediated suppression of POMC transcription (Figure 2C). Consistent with the results obtained with POMC-Luc, CIRP increased basal ACTH secretion, reversing the effects of dexamethasone on endogenous ACTH secretion in AtT20 cells (Figure 2D). Physiologically, hypothalamus-released CRH stimulates ACTH secretion by activating CRH receptors located in pituitary corticotroph cells. Next, we further examined the synergistic effect of CRH and CIRP on ACTH secretion in AtT20 cells. AtT20 cells overexpressing CIRP were treated with or without 100 nM CRH for 4h or 24h. It showed that both CRH and CIRP individually stimulated ACTH secretion, and there was further increase in ACTH secretion when they were combined (Figure S3A). In addition, the mRNA expression level of CRHR1 was increased by 40% by overexpressing CIRP, measured by qRT-PCR (Figure S3B).

### CIRP promote cell proliferation and clonogenic ability of AtT20 cells

Next, we tested the effects of CIRP on AtT20 cell proliferation. Cell proliferation was enhanced in a time-dependent manner (ANOVA; *P* < 0.05) in AtT20 cells stably transfected with CIRP, with significant effects observed from 24h (20.0%; *P* < 0.05) to 120h (49.4%; *P* < 0.001) (Figure 2E, 2F). In addition, as compared with vector control, CIRP overexpression contributed to the formation of colonies of AtT-20 cells (*P* < 0.01) (Figure 2G).

### CIRP increases corticotroph tumor growth and ACTH secretion *in vivo*

**Figure 3:**
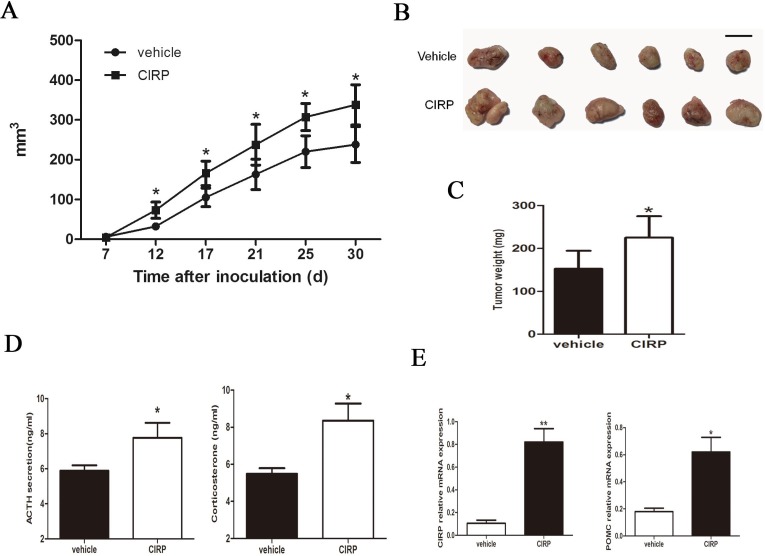
CIRP promotes murine corticotroph cell growth *in vivo* **A.** tumor growth rate; **B.** tumor depiction; **C.** tumor weight; **D.** circulating ACTH and corticosterone levels derived from mice harboring stable CIRP overexpressing cells compared with controls. **E.** qRT-PCR confirmed increased CIRP and POMC mRNA in stable CIRP transfectant tumor tissues compared to controls. *P < 0.05, **P < 0.01.

Based on these findings, we next examined the effects of CIRP on corticotroph tumor xenografts generated by inoculating AtT20 cells stably overexpressing CIRP in 6-week-old nude mice. As shown in Figure 3A and 3B, compared with vehicle-treated control tumors, CIRP overexpression significantly enhanced tumor growth for the duration of the experiment (Figure 3A, 3B). Tumors derived from CIRP-transfected corticotroph tumor cells were significantly greater than those from the control group (83.1±62.5 *vs* 32.2±26.0 mm^3^; Student's t test, *P* < 0.05) as early as 12 days after inoculation. Thirty days after inoculation, the average tumor volume of CIRP overexpression mouse was 142% of that of controls (238.2±127.4 mm^3^ *vs* 363.0±111.1 mm^3^; Student's t test, *P* < 0.05). Furthermore, larger tumor weights were observed in mice harboring CIRP-transfected AtT20 cells (225.1 ± 49.8 *vs* 152.3 ± 42.0 mg, *P* < 0.05) (Figure 3C). Consistent with the faster tumor growth rate and development of larger tumors in mice inoculating with CIRP-transfected cells, plasma ACTH and serum corticosterone levels were also 1.3 and 1.7 fold higher, respectively, as compared to controls (Figure 3D). Additionally, higher CIRP and POMC expression was detected in tumors formed from CIRP overexpressing cells compared with control tumors (Figure 3E).

### Knockdown of CIRP inhibits the proliferation of AtT20 cells *in vitro*

**Figure 4:**
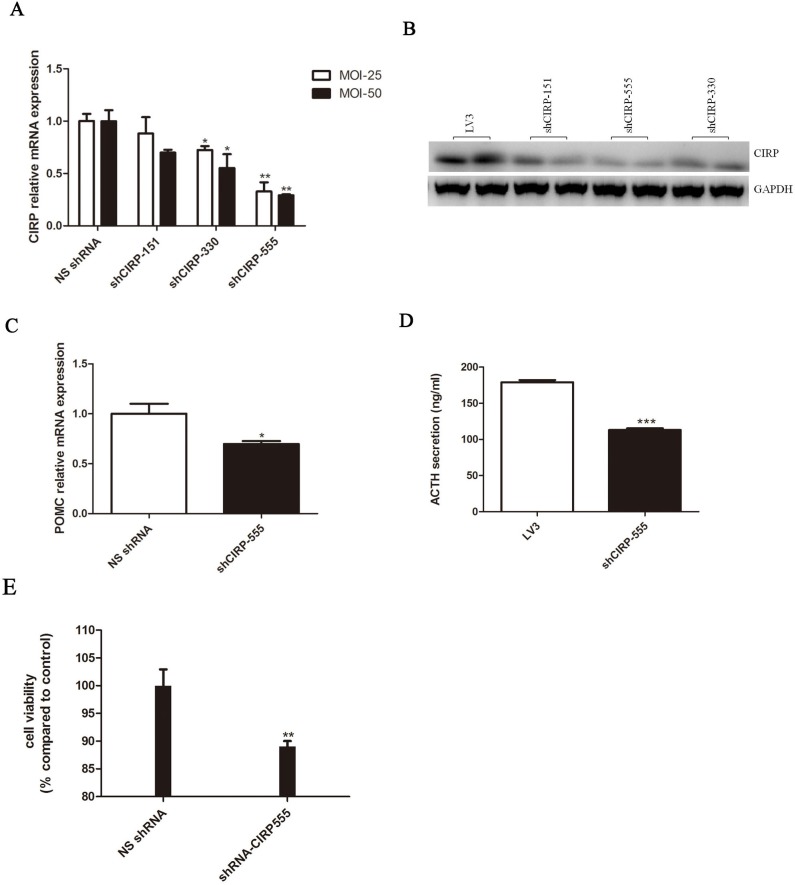
CIRP knockdown inhibits ACTH secretion and cell proliferation in AtT20 cells **A.** Effect of CIRP knockdown on POMC mRNA expression. **B.** Secreted ACTH levels in vehicle or CIRP knockdown AtT20 cells. **C.** Effects of CIRP knockdown on AtT20 cells proliferation.

To further verify that CIRP expression was related to CD progression, three shRNAs (shCIRP-151, shCIRP-330, shCIRP-555) were designed to silence CIRP expression in AtT20 cells. shCIRP-555, reducing the levels of CIRP to approximately 69% of control (Figure 4A, 4B), is the most effective one and is chosen for subsequent studies. Transfection with shCIRP-555 resulted in a markedly decrease in POMC mRNA expression and a 36.8% decrease in ACTH secretion (Figure 4C, 4D). In addition, shRNA transfection-mediated CIRP knockdown inhibited cell growth compared with negative control cells (Figure 4E).

### Effects of CIRP on cell proliferation are mediated by p27 and cyclinD1 *via* Erk1/2

**Figure 5:**
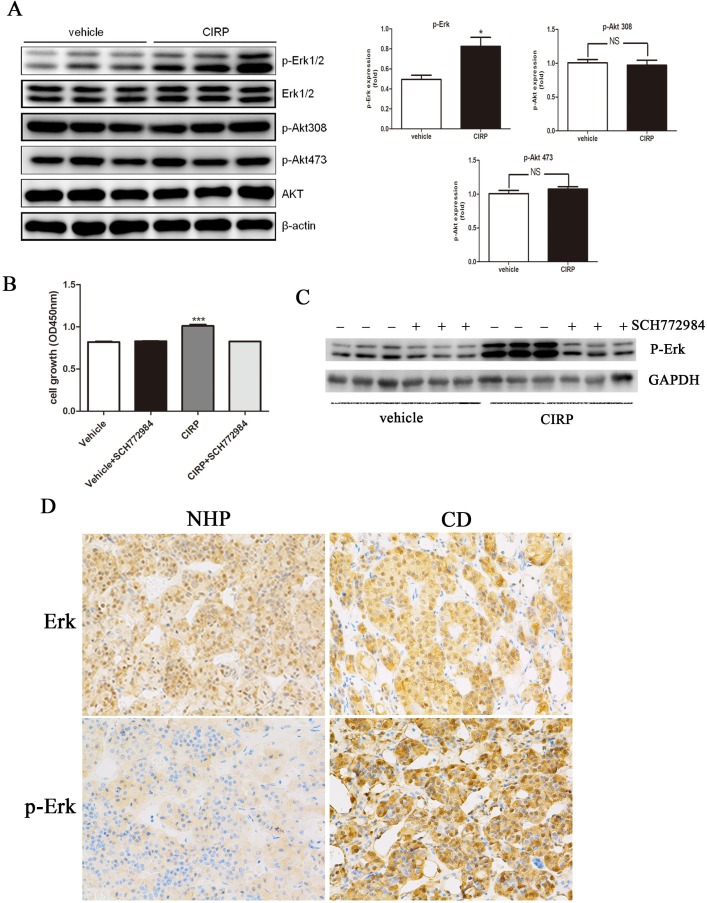

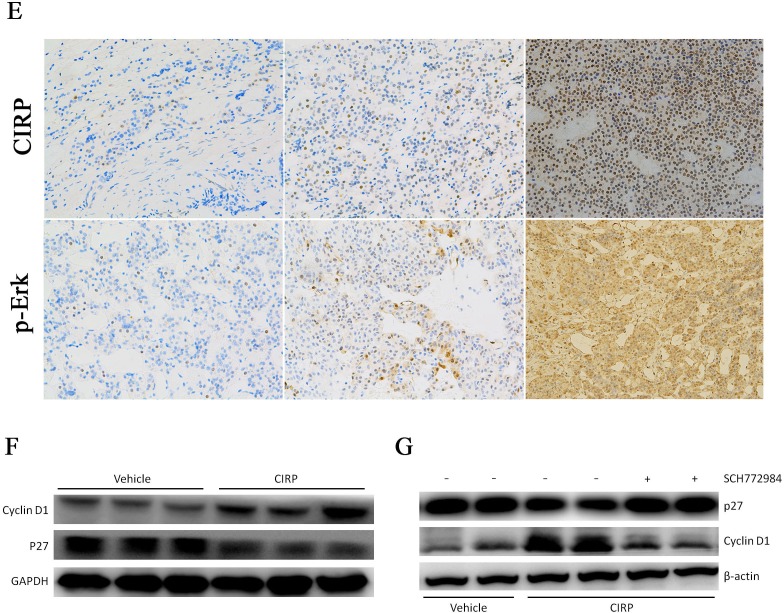
CIRP could upregulated cyclinD1 and downregulated p27 level *via* Erk signaling pathway **A.** CIRP overexpression promotes phosphorylation of Erk in AtT20 cells, while it had no effect on phosphorylation state of Akt. **B.** Blockade of Erk activation with SCH772984, the cell proliferation was not influenced by CIRP. **C.** After SCH772984 treatment, the Erk activity was significantly inhibited. **D.** Example of the p-Erk immunoblot in normal human pituitary (NHP) and one CD patient where CIRP was upregulated. **E.** Representative images of concordant expression of CIRP and p-Erk. Left, middle and right panels depict mild, moderate and abundant staining of CIRP (upper) and p-Erk (lower), respectively. Original magnification, ×200. **F.** cyclinD1 protein level was elevated and p27 level was decreased after CIRP overexpression. **G.** Blockade of Erk activity with SCH772984, cyclinD1 and p27 levels was not affected by CIRP.

After CIRP overexpression, western blotting analysis showed that phosphor-Erk protein levels were obviously increased, whereas phosphor-Akt protein level was not significantly changed (Figure 5A). Furthermore, blockade of Erk1/2 activation with a specific inhibitor SCH772984, the effect of CIRP on cell proliferation and p-Erk expression was significantly reversed (Figure 5B, 5C). In order to determine if CIRP upregulation might be associated with an increase in p-Erk1/2 status in CD, p-Erk1/2 was analyzed in six CD samples and six normal human pituitaries tissues. p-Erk1/2 was upregulated in four of six samples by immunohistochemical analysis, as compared with that in normal pituitary tissue. An example of six patients is shown in Figure 5D. In order to determine if this association was truly significant, we assayed a larger sample of corticotroph tumors (25 patients) using immuohistochemistry for CIRP and p-ERK1/2 antibodies. Furthermore, this result was supported by the facts that patients with mild CIRP staining exhibited weak phospho-Erk immunoreactivity, with moderate CIRP signals showed moderate phospho-Erk expression, and with strong CIRP signals exhibited abundant phospho-Erk protein expression (Figure 5E).

Previous studies have suggested a role of p27 and cyclinD1 in tumorigenesis of CD. Moreover, p27 protein level was decreased and cyclinD1 level was increased in CD (Figure S4). In AtT20 cells overexpressing CIRP, expression of p27 was decreased and that of cyclinD1 was induced (Figure 5F). Because p27 and cyclinD1 are known to be phosphorylated by Erk1/2 [17], we examined whether the effects of CIRP on p27 and cyclinD1 depended on Erk1/2. We inhibited Erk1/2 activity with SCH772984 in AtT20 cells overexpressing CIRP. When the activity of Erk1/2 was inhibited, the level of p27 and cyclinD1 was not affected by CIRP either (Figure 5G).

## DISCUSSION

In mammals, only two cold shock proteins have been described in detail to date, CIRP and RNA-binding motif protein (RBM3) [9]. In general, mammalian cells rarely reach temperatures close to 32°C *in vivo*, although there are some exceptions for certain organs, such as testis or the skin. Thus, CIRP must exert additional functions at physiological and pathological conditions that have not been fully explored. There is now growing evidence that in addition to their role in cold stress responses, they also play critical roles in cancer cell survival and growth [18, 19]. In this study, we identified a novel role of CIRP in CD. We demonstrated that CIRP expression is significantly up-regulated in corticotroph adenoma. CIRP could regulate POMC gene expression and ACTH secretion *in vitro*, and elevated CIRP levels enhanced the secretion of ACTH and corticosterone *in vivo*. Moreover, CIRP expression promoted corticotroph tumor growth and proliferation *in vitro* and *in vivo*. Furthermore, knockdown CIRP with shRNA could inhibit ACTH secretion and cellular proliferation. Our results indicated that CIRP might be implicated in the Cushing's tumorigenesis.

It is noteworthy that higher CIRP expression was also noted in nonfunctional pituitary adenoma. Whether CIRP is also involved in the pathogenesis of non-functioning adenoma is deserved to be further explored. Compared with that in normal pituitary tissue, CIRP expression level in somatotroph and lactotroph adenoma was not obviously changed. Human corticotroph tumors showed increased invasiveness and recurrence compared with other hormone-secreting pituitary tumors [20], which indicates that corticotroph cells are more proliferative than other cell types. It would, therefore, seem that high CIRP expression is a characteristic of corticotrophs that may reflect their intrinsic proliferative rate, although there is no direct evidence in support of this speculation. In addition, no molecular marker predictive for CD relapse has been reported so far. Clinically, we showed that patients with higher CIRP expression are more likely to recur than those with rare or no CIRP expression; Moreover, in postoperative recurrent patients, CIRP expression levels in sample from secondary surgery were remarkably higher than that from the initial one, which indicated that CIRP is correlated with recurrence. This result is consistent with the finding reported in a recent study that recurrent pituitary adenoma expressed significantly higher CIRP levels compared to non-recurrent tumors and CIRP expression is correlated with tumor proliferation and invasion [21]. CIRP, thus, might be a marker for predicting the risk of CD recurrence. However, a future larger scale study of CIRP in patients with corticotroph adenoma will be critical to determine whether high CIRP expression is able to predict the risk of CD recurrence.

The mammalian Erk-MAPK pathway is implicated in multiple physiological processes, including cell proliferation, differentiation and survival [22, 23]. The activation of Erk1/2 induces proliferative signals that may contribute to tumorigenesis or cancer growth, as seen by the presence of activated Erk1/2 in a variety of human cancer tissues and cell lines, including pituitary adenoma [24-27]. Thus, we examined whether the effect of CIRP on AtT20 cell proliferation was mediated by stimulating the Erk pathway. Our results showed that CIRP expression led to an increase in the phosphorylation state of Erk1/2 kinase in AtT20 cells, while it had no effect on the phosphorylation state of Akt, demonstrating that CIRP has a specific mode of action through the Erk1/2 signaling pathway. In order to unravel the intricate mechanism occurring downstream of p-Erk1/2, several cell cycle regulatory proteins were studied. p27^kip1^, a cyclin-dependent kinase inhibitor, is down-regulated in corticotroph adenoma [28]; p27 knockdown mice developed pituitary tumors that immunostain for ACTH and p27 germline mutations are associated with human corticotroph adenomas [29, 30], which indicated a key role for p27 depletion in corticotroph tumorigenesis. CyclinD1 is a proto-oncogene which acts on G1-S progression of the cell cycle. The expression of cyclinD1 was elevated in pituitary adenomas *versus* those in the normal pituitary gland and cyclinD1 overexpression is associated with recurrence [31, 32]. Immunoblotting analysis revealed that CIRP overexpression caused a significant increase in cyclinD1 protein levels and decrease in p27 protein levels; furthermore, blockade of Erk activation with a specific inhibitor SCH772984, the level of cyclinD1 and p27^kip1^ was not affected by CIRP either. These data demonstrate that expression of CIRP promote proliferation by inducing the kinase activity of Erk1/2 toward p27 and cyclinD1 in AtT20 cells.

Discovery of CIRP promoting of cell proliferation and ACTH secretion not only advances our understanding of pathogenesis of CD but will also help in the development of new therapeutic strategies. However, further research is urgently needed to clarify the exact functions and the underlying mechanisms of CIRP in the pathogenesis of corticotroph adenoma in humans.

In summary, in the present study we showed that CIRP was dysregulated in ACTH-secreting pituitary adenomas. In addition, we demonstrated that CIRP exerts its effects by regulating Erk-MAPK signaling pathway. Suppression and measurement of CIRP expression is a promising approach for advanced treatment and management of CD patients.

## MATERIALS AND METHODS

All reagents were purchased from Sigma-Aldrich unless stated otherwise.

### Human tissue samples

The human normal pituitaries (*n* = 7, 3 males, 22-67 years old) were obtained from fresh autopsy specimens. And human pituitary tumor samples were obtained by transsphenoidal surgery from patients with pituitary adenoma, including 45 cases of Cushing's disease, 8 cases of acromegaly, 6 cases of prolactinoma and 7 cases of nonfunctioning adenoma. The clinical diagnosis of individual tumors was established on the basis of clinical features, endocrine assessment, MR imaging, histology and pituitary hormone immunohistochemistry. This study was approved by the Ruijin Hospital Ethics Committees and the written informed consent was obtained from all patients.

### Cell culture and stable cell transfections

The mouse AtT20 pituitary corticotroph cell line was obtained from the American Tissue Type Collection (ATCC, Manassas, VA, USA). The cells were maintained in Dulbecco's modified Eagle's medium (DMEM) containing 10% fetal bovine serum (FBS) and 2mM L-glutamine and 100 μg/ml penicillin/streptomycin (GIBCO, Carlsbad, CA, USA) in a humidified incubator with 5% CO_2_ at 37°C. pMSCVpuro Vector (Clontech, No.634401) was used. Puromycin (100 ng/ml) was added to AtT20 transfectants for 14 days to select stable colonies presence of pMSCVpuro vector.

### Immunoblotting

Total cell lysate was prepared with RIPA buffer containing Protease Inhibitor Cocktail. Protein concentrations were measured by DC protein assay reagent (Bio-Rad) and extracts resolved by SDS/PAGE on 8% gels. Membranes were blocked for 2 h at room temperature in TBS-Tween-20 containing 5% nonfat dried milk (Bio-Rad), washed, and then incubated with primary antibodies (anti-CIRP from Santa cruz; anti-pErk1/2, anti-Erk1/2, anti-Akt, anti-pAkt, anti-β-actin, anti-p27/kip1, anti-cyclinD1 from Cell Signaling Technology) at 4°C overnight. After washing, membranes were incubated with HRP-conjugated secondary antibodies (Cell Signaling Technology). The signal was detected using enhanced chemiluminescence (PerkinElmer, Waltham, MA, USA).

### Quantitative real time-PCR (qRT-PCR)

The total RNA was extracted with TRIzol reagent (Invitrogen, Carlsbad, CA). For reverse transcription, 1 μg of the total RNA was converted into cDNA in a 20 μl reaction volume using a reverse transcription kit (Promega) following the manufacturer's instruction. Real-time PCR was carried out on the LightCycler 480 system (Roche) using SYBER Green Supermix (Takara). The relative RNA levels were calculated on the basis of 2^CT^ and normalized to GAPDH mRNA levels. mRNA expression levels of all genes were normalized to GAPDH after confirming that its mRNA are unaffected by CIRP treatment. Quantification was performed in quadruplicate, and the experiments were repeated independently three times. The sequences of the primers were as follows (Table 2).

**Tabel 2:** Primers sequence

species	Gene name	Primer sequence (5′-3′)
Human	CIRP	F: AGGGCTGAGTTTTGACACCAA
		R: ACAAACCCAAATCCCCGAGAT
Mouse	CIRP	F: GGACTCAGCTTCGACACCAAC
		R: ATGGCGTCCTTAGCGTCATC
Mouse	POMC	F: CCACTGAACATCTTTGTCCCCA
		R: GCATCTTCCACGTGTCAGGC
Mouse	CRHR1	F: CGCATCCTCATGACCAAACTC
		R: CGCATCCTCATGACCAAACTC

### Hormone assays

After the cells were incubated for the specified time interval, the culture medial from each well was collected for further measurement. The ACTH in the culture media was measured using ACTH (rat, mouse) ELISA kit (Phoenix, Milpitas, USA). The samples in each experimental group were analysed in quintuple and the experiments were repeated independently three times.

### Luciferase reporter assays

AtT20 cells were plated at 1×10^5^ cells per well in 24-well plates in cell medium contain 10% FBS and left them to attach for 24h. In each well, we transfected 0.5 μg CIRP and 0.5μg POMC-Luc reporter gene plus 0.25ng SV40 for normalization for 48h using Lipofectamine 3000 according to the manufacturer's instructions. AtT20 cells were collected 48 hours after transfection and analyzed using the Dual-Luciferase Reporter Assay System (Promega).

### Cell proliferation assay and colony formation test

AtT20 cells were seeded into 96-well plates at 2000 cells/well and incubated for 24 h to attach. Cell viability was determined using Cell Counting Kit-8 assay (Dojindo Laboratories, Kumamoto, Japan) according to the manufacturer's protocol, or cells were counted at the indicated time points.

AtT20 cells were plated at a density of 10^3^ cells/well in a 6-well plate in DMEM culture medium containing 10% FBS. The medium was replaced every 4 or 5 days. After 21 days, colonied were fixed with 4% paraformaldehyde in PBS containing 4% sucrose for 20 min, and then stained with 0.005% crystal violet for 30 min at room temperature. Then, they were washed three times with PBS for 5 min. Colonies containing more than 50 cells were counted using an Axiovert inverted microscope.

### Immunocytochemistry and immunohistochemistry

AtT20 cells were grown on coverslips, fixed with 4% paraformaldehyde for 30 min, and permeabilized with 0.5% Triton X-100 for 20 minutes. Then coverslips were blocked with 2% rabbit serum for 1 h and hybridized with antibody against CIRP (1:200, Abcam) overnight at 4°C. Then the cells were washed in PBS and incubated with FITC-conjugated (1:200, Proteintech, Chicago, IL) goat anti-rabbit IgG, avoiding light for 1 hour. Finally, the cells were dyed by DAPI with blue fluorescence for 10 min. The images were acquired using an Olympus microscope.

The tissues were fixed in 4% paraformaldehyde for at least 24h, dehydrated and paraffin embedded. Hematoxylin-eosin staining was performed on 4-μm sections. Immunohistochemical staining was performed according to the standard protocols. The following primary antibodies were used: anti-CIRP antibody (1: 200; Abcam); anti-ACTH antibody (1: 200; Abcam). The images were acquired using an Olympus microscopy system. The IHC staining intensity was scored as previously reported [15], that is, tumors were considered positive if more than 10% of the cells showed moderate to strong staining.

### Xenograft transplantation and *in vivo* studies

Six-week-old nu/nu male mice were inoculated subcutaneously with empty vector or CIRP overexpressing stable transfectant AtT20 cells (5×10^5^ cells per mouse). Each group comprises 10 mice, and tumor size was measured by caliper measurements every 4-5 days, and the volume was calculated with the formula 1/2 × (length×width^2^). Thirty days after cell inoculations (each group comprises 8 mice), animals were euthanized and tumors were excised and weighted. Blood samples were collected on the day of euthanization. All of the animal procedures were conducted in accordance with the Guide for The Care and Use of laboratory Animals published by the National Institutes of Health.

### Statistics

The statistical analysis was performed using SPSS version 16.0. Values are described as mean ± standard deviation. Significant differences were analysed using two-tail unpaired Student *t* test and one-way ANOVA; the Mann-Whitney U-test were used for the continuous variables. The *P* value less than 0.05 was considered statistically significant.

## SUPPLEMENTARY MATERIAL FIGURES


