# Supplementary material for: Cold inducible RNA binding protein upregulation in pituitary corticotroph adenoma induces corticotroph cell proliferation via Erk signaling pathway
Source: Oncotarget. 2016 Jan 27;7(8):9175–87. doi: 10.18632/oncotarget.7037 (PMC4891034; doi:10.18632/oncotarget.7037)
Supplement: Supplementary file 1 [file oncotarget-07-9175-s001.pdf]

Cold inducible RNA binding protein upregulation in pituitary corticotroph adenoma induces corticotroph cell proliferation via Erk signaling pathway

Supplementary Material

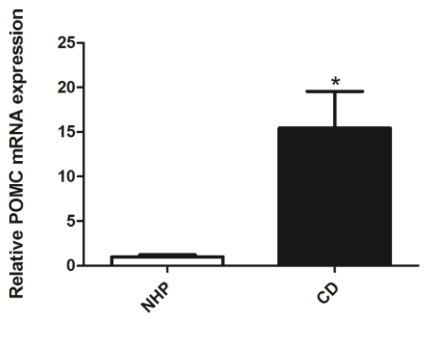

Fig. S1 q-PCR analyses of POMC mRNA expression in normal human pituitary (NHP) and Cushing's disease (CD).

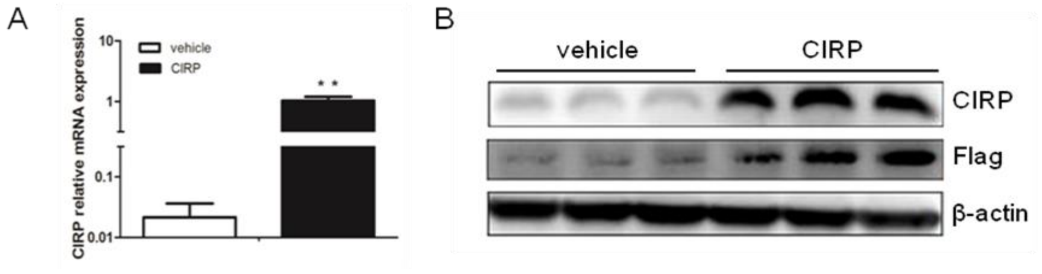

Fig. S2 Efficacy of pMSCV-CIRP overexpression. qPCR and Western blot analyses of CIRP (and flag) expression in CIRP overexpression or vehicle-treated AtT20 cells.

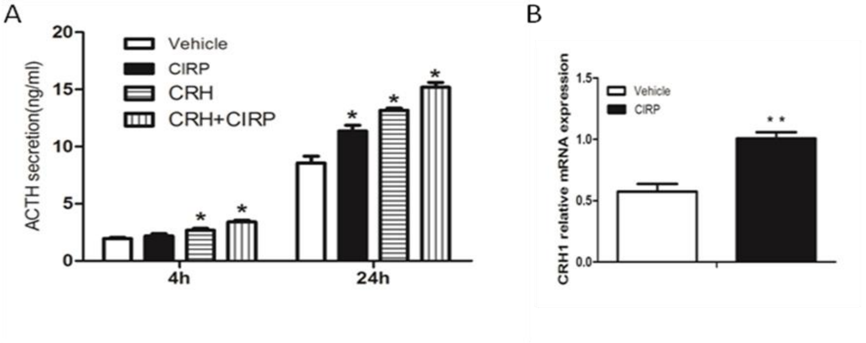

Fig. S3 A, Secreted ACTH levels with and without CRH stimulation in vehicle or CIRP-overexpressing AtT20 cells. B, qRT-PCR of CRHR1 mRNA.

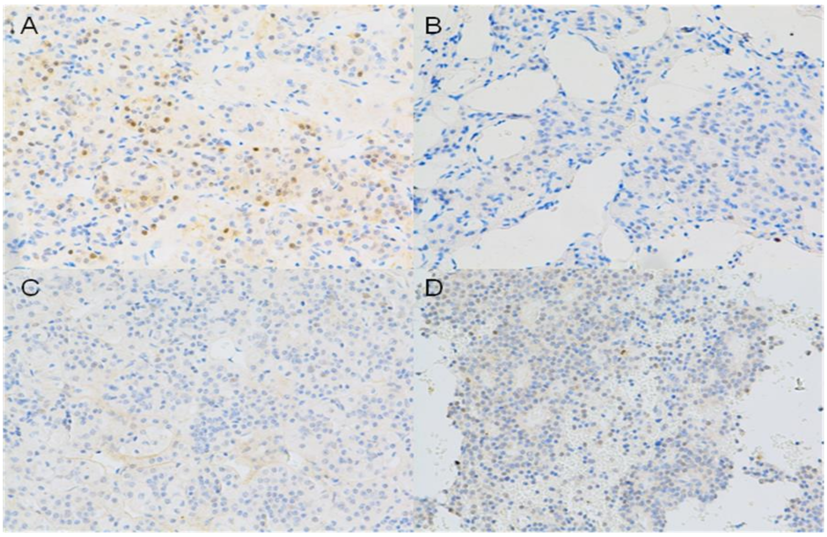

Fig. S4 Immunostaining for P27 (A and B) and cyclin D1(C and D) in normal human pituitary (left) and corticotroph adenoma (right). Original magnification, ×200.
